# Tumor lysis syndrome in an extraskeletal osteosarcoma: a case report and review of the literature

**DOI:** 10.1186/s13256-017-1241-3

**Published:** 2017-03-24

**Authors:** Vito Emanuele Catania, Michele Vecchio, Michele Malaguarnera, Roberto Madeddu, Giulia Malaguarnera, Saverio Latteri

**Affiliations:** 1 Department of Medical-Surgical Sciences, and Advanced Technologies “G. F. Ingrassia”, University of Catania, via Santa Sofia 86, 95123 Catania, Italy; 2 UOC Centre of Physical Medicine and Rehabilitation, AOU “O.V.E-Policlinico”, Catania, Italy; 3 Research Centre “The Great Senescence”, University of Catania, Catania, Italy; 4 Department of Biomedical Sciences, University of Sassari, Sassari, Italy

**Keywords:** Tumor lysis, Osteosarcoma, Case report, Uricemia, Kalemia, Rasburicase

## Abstract

**Background:**

This case report describes a spontaneous tumor lysis syndrome due to a rare solid tumor.

**Case presentation:**

A 65-year-old white woman had tumor lysis syndrome, which represent a dangerous oncological emergency. This syndrome occurs usually with a hematological tumor, but in this case our patient had a solid tumor, which was a rare extraskeletal osteosarcoma, localized in her pelvic region. She also had lung metastases and bilateral hydronephrosis.

After spontaneous tumor lysis syndrome, she had acute renal insufficiency, which was treated with hemodialysis and successively with rasburicase, Kayexalate (sodium polystyrene sulfonate), and febuxostat.

**Conclusion:**

Tumor lysis syndrome represents an oncological emergency, which must be suspected and treated as soon as possible.

## Background

Tumor lysis syndrome (TLS) is one of the major oncological emergencies; disruption is caused by massive tumor cell lysis in which the contents of the lysed tumor cells are released into the bloodstream [1]. It is a potential fatal complication of the treatment of hematological tumors and, less frequently, of solid aggressive tumors. Hematologic malignancies comprise the vast majority of TLS, which is believed to be secondary to treatment sensitivity and rapid proliferative rates [2, 3]. Patients with a solid tumor usually show TLS after intensive chemotherapy (treatment-induced TLS), but sometimes it can appear before the treatment (spontaneous TLS) especially in cases of bulky tumors with wide necrotic areas. TLS can alter the body’s homeostatic mechanisms and cause hyperuricemia, hyperkalemia, hyperphosphatemia, and uremia [4].

Extraskeletal osteosarcoma (ESOS) is a rare subtype of osteosarcoma without attachment to bone or periosteum. It accounts for less than 4% of all osteosarcomas and approximately 1 to 2% of all soft tissue sarcomas (STSs) [5]. The clinical features of patients with this tumor differ significantly from the clinical features of patients with skeletal osteosarcoma, including older age, propensity for axial tumors, and female preponderance.

Although ESOS has been found to develop in all organs, the most common locations are limbs. In the case of abdominal or pelvic lesions the diagnosis can be very difficult, thus it requires confirmation after exploratory laparotomy and histopathology. Such tumors may reach an enormous size before detection, because the enlarging mass may not be associated with pain. ESOS may be one of the differential diagnoses to be considered in the case of calcified masses arising in retroperitoneal space [5, 6].

In previous studies, the total number of patients reported with both tumor lysis and sarcoma was five: three males and two females and their age spanned from 9 years to 66 years. All of them had evidence of advanced and metastatic disease [5–10]. One patient was reported to have normal baseline values of creatinine and phosphorus, whereas another patient had elevated baseline values of creatinine and lactate dehydrogenase (LDH). Three cases were preceded by chemotherapy; the other two cases were represented by spontaneous TLS. All the cases taken in consideration had baseline elevation of uric acid and phosphorus.

Clinical tumor lysis occurred more frequently in patients with pre-treatment renal insufficiency than in patients with normal renal function. The development of uremia is correlated with high pre-treatment serum LDH concentrations [4].

## Case presentation

A 65-year-old white woman was hospitalized for a right hypochondrium colicky pain radiating to the ipsilateral subscapularis region. Her pain was not related to food and posture. It appeared during the previous week and persisted despite nonsteroidal anti-inflammatory medication. A clinical examination showed that her abdomen was swollen in the right iliac fossa and the presence of voluminous palpable mass that was hard, fixed to the deep layers, and sore to touch.

An abdominal ultrasound (US) examination revealed her liver with gallbladder stones, a heterologous formation in her right iliac fossa with internal calcification, which compressed the iliac vessels, and a bilateral hydronephrosis more marked on the right than on the left.

Laboratory tests revealed significant alterations (Table 1). Her blood pressure was above 180 mmHg. A chest X-ray showed the presence of multiple metastases placed in both lung fields. A total body computed tomography scan (CT) without contrast confirmed the presence of lung metastases, bilateral hydronephrosis, and a mass in her right iliac fossa invading her ureter and her iliac artery.

**Table 1:** Laboratory tests before and after hemodialysis

Laboratory tests	Before dialysis	After dialysis	Reference range
Alkaline phosphatase IU/l	1460	670	20–140
Urea nitrogen mg/dl	196.2	58.3	10–50
Creatinine mg/dl	7.89	1.36	0.60–1.50
Phosphorus mg/dl	4.9	4.2	2.6–4.5
Calcium mg/dl	7.5	7.7	8.5–10.5
Sodium mmol/l	144	136	135–145
Potassium mmol/l	6.1	4.2	3.4–4.8
Lactate dehydrogenase U/l	1120	870	110–210
Uric acid mg/dl	11.4	6.7	3.6–8.5

There was also a mass in the right iliac mass but with a reduced size in the left iliac fossa invading her psoas muscle. The internal structure of the masses was homogeneous, apart from the centrally located amorphous high-attenuation areas. There was no fat inside the lesion.

She underwent hemodialysis and infusion therapy with rasburicase (Fasturtec ®; Table 1).

Prompt dialysis is necessary when treatment fails to normalize electrolytes or establish urinary flow. Hemodialysis removes excess circulating uric acid. Before the dialysis our patient was hyperuricemic and oliguric. After the dialysis, we observed that she developed diuresis, associated with the consequent resolution of hyperuricemia, hyperazotemia, hyperphosphatemia, and normalization of electrolytes.

After 10 days she underwent surgery. An exploration of her abdominal cavity detected a retroperitoneal mass in her right iliac region which involved the last ileal loop besides compressing the iliac vessels and infiltrating her ureter. Another mass in her left iliac fossa caused a compression of the left iliac vessels and ureter without infiltration. As a result of the inoperability of the mass in her right iliac region, an ileotransversostomy was performed with enucleation of the left mass and cholecystectomy.

**Fig. 1:**
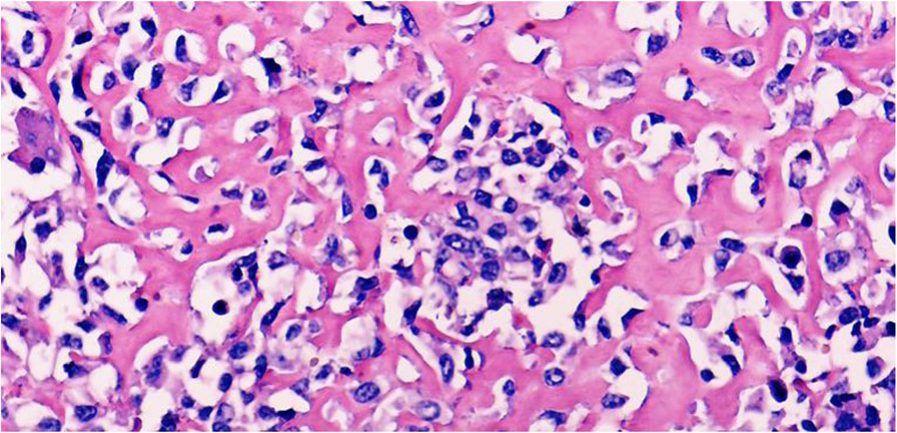
Round and multinucleated cells separated by osteoid material (×200)

An histological examination performed on the mass diagnosed an ESOS infiltrating her psoas muscle, her internal iliac artery, and her bladder wall. On macroscopic examination the mass had a hard consistency, was whitish-gray in color, and there were large central calcified parts and areas of hemorrhagic-necrotic tissue. The mass was osteoblastic histological type, consisting of osteoblasts producing an osteoid material. The lesional cells showed great cytological atypia, high mitotic activity, and permeative growth pattern (Fig. 1).

The tumor massively invaded our patient’s abdominal cavity and metastasized in both lung fields. According to these parameters, she was in a very poor prognostic subgroup, due to a very bulky and high growth rate tumor. This tumor was only partially resectable because she was over 60-years old and showed multiple metastatic lesions, multiorgan impairment, and increased alkaline phosphatase (ALP) levels. A CT scan showed large soft tissue masses with focal or massive areas of calcification and no osseous involvement. Magnetic resonance (MR) images showed intermediate signal intensity on T1-weighted images and a low-to-hyperintense signal on T2-weighted images.

The diagnosis of osteoblastic ESOS was confirmed by the tumor localization within the soft tissue, without attachment to bony structures, and the presence of abundant osteoid. There was no history of radiation or trauma in our patient, but there was an anamnestic finding of hysterectomy just 6 months before the diagnosis of ESOS.

The most common presentation of ESOS is a gradually enlarging mass that varies in size from 1 to 50 cm in diameter and does not always imply pain. Very large and bulky tumors often develop in the retroperitoneum before their detection as in our case. Thus the presence of metastatic lesions, the patient’s age, and the size were the major prognostic factors; in addition, patients with very large lesions have a worse clinical outcome.

## Discussion

ESOS is a high-grade malignant mesenchymal neoplasm with very aggressive behavior. The tumor is typically located in the deep soft tissues, without attachment to skeletal bones. The most common affected site is the thighs, followed by upper extremities, retroperitoneum, and buttocks.

The etiology of ESOS is essentially unknown, even though there have been reported cases of radiation-induced ESOS, a history of previous trauma, intramuscular injection at the tumor site, and malignant transformation of myositis ossification. ESOS has also been shown to develop in unusual sites, such as mediastinum, hand, cerebellum, heart, skin, pleura, larynx, scalp, tongue, penis, gallbladder, breast, and mesentery.

The treatment of choice for ESOS is amputation or a wide surgical resection of the tumor for local control and to prevent a possible recurrence. Chemotherapy and radiation therapy can be combined to surgery although their role in neoadjuvant or adjuvant therapy has not been clearly defined yet. Nevertheless, classical high-grade ESOS still remains a very aggressive rare tumor with a poor prognosis.

Our patient had a spontaneous TLS, with an acute renal insufficiency, treated with hemodialysis (Table 1). After this treatment, TLS was treated with rasburicase, Kayexalate (sodium polystyrene sulfonate), abundant hydration, and febuxostat (Fig. 1) [11, 12].

Risk factors for TSL include: (1) presence of bulky disease, particularly with pluri-metastatic sites (especially abdominal metastases), bulky adenopathy, and hepatosplenomegaly; (2) high leucocyte count; (3) elevated pre-treatment LDH; (4) elevated pre-treatment uric acid; (5) compromised renal function; and (6) history of use of potentially nephrotoxic drugs [7].

The most frequent serological alteration is: hyperuricemia, hyperkalemia, hyperphosphatemia, and hypocalcemia. Both hyperuricemia and hyperphosphatemia can evolve in renal failure and they can also cause cardiologic complications. The contemporaneous presence of the two electrolytic alterations may cause a fall in cardiologic function [8–10]. Hyperuricemia results from the release and breakdown of purine nucleic acids. Uric acid is poorly soluble in water and, when normal limits of excretion are surpassed, precipitation of crystals occurs in the renal tubules leading to renal insufficiency and, potentially, failure [13].

Malignant cells often have a higher phosphate concentration than normal cells. Rapid lysis can lead to hyperphosphatemia when the normal homeostatic mechanisms are overwhelmed. Excretion of phosphate may be further impaired because of acute kidney injury either from uric acid crystal deposition or from tumor involvement of the kidneys [14]. In the setting of hyperphosphatemia, calcium phosphate precipitation may also occur in renal tubules, contributing further to acute kidney injury [15]. Hyperphosphatemia may also cause nausea, vomiting, diarrhea, lethargy, or seizures. Hypocalcemia in TLS is a consequence of hyperphosphatemia, causing calcium phosphate precipitation in tissues.

TLS may be clinically asymptomatic, but hyperkalemia and hypocalcemia may cause neurologic, cardiovascular, or muscular symptoms including seizures, arrhythmias (typically with a longer QT space), and tetany. Hyperkalemia is a potentially life-threatening consequence of TLS, occurring when large quantities of potassium are released from necrotic tumor cells. Acute kidney injury, if present, can worsen hyperkalemia. A rapid increase in serum potassium levels may result in cardiac arrhythmia and cardiac arrest [16–18].

In neoplastic patients, acute tumor lysis may be a cause of acute renal failure (ARF) due to severe metabolic derangement. Acute TLS has rarely been investigated and most patients developed spontaneous and oliguric ARF before therapy. The cause of renal failure is now recognized as acute uric acid nephropathy, due to increased uric acid production and hyperuricosuria resulting from a high tumor cell turnover rate. A key distinction between spontaneous tumor lysis and TLS is the lack of hyperphosphatemia in the spontaneous form occurring after therapy.

### Search strategy

We searched PubMed/Medline, Scopus, Embase, and Web of Sciences articles that focused on TLS in patients with osteosarcomas published from 1960 to September 2016. The search methodology was adapted from scientific search guidelines published in 2011.

## Conclusions

Previous studies have shown a reduced incidence of significant post-treatment hyperuricemia and hyperphosphatemia as the most common laboratory abnormalities associated with post-treatment ARF. Prospective investigations are required to compare conservative treatment with pre-emptive dialysis and to develop reliable clinical profiles identifying patients at risk of developing renal failure and increasing the potential of TLS.

**Fig. 2:**
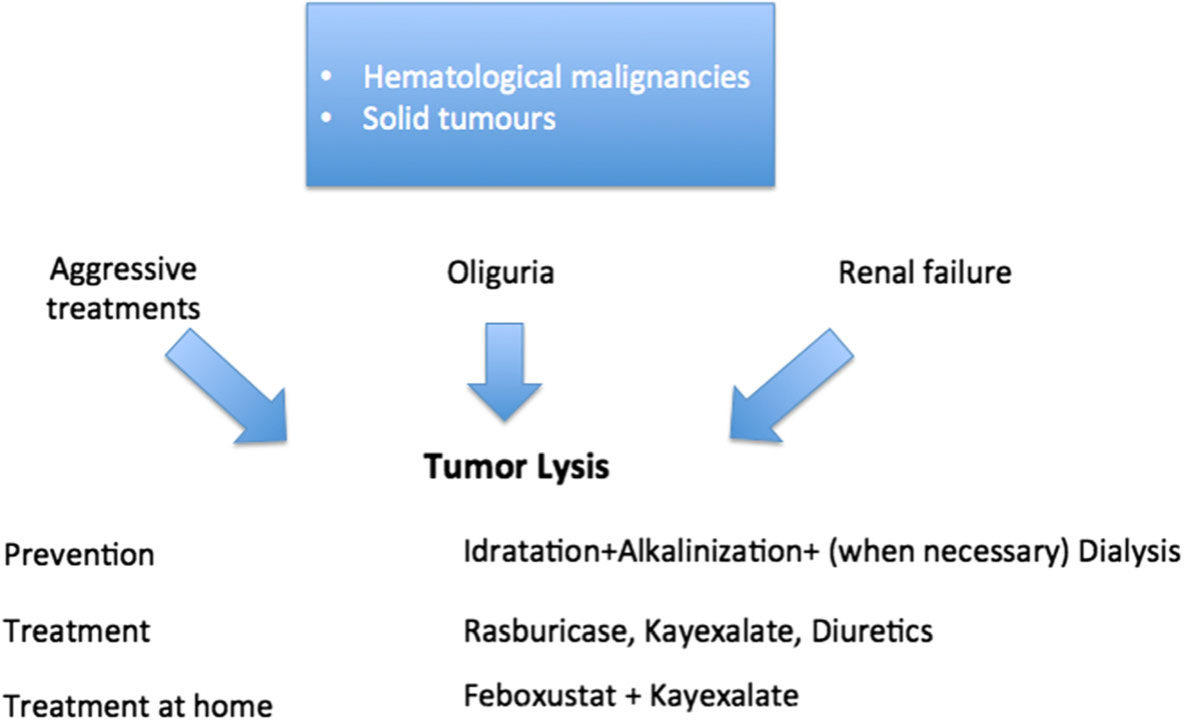
Cause, prevention, and treatment of tumor lysis syndrome

Acute TLS often represents a fatal complication in treated and non-treated tumors. A prompt treatment of TLS in ESOS and in others tumor reduces complications and can improve prognosis and quality of life (Fig. 2).

## Abbreviations

ALP: Alkaline phosphatase
ARF: Acute renal failure
CT: Computed tomography
ESOS: Extraskeletal osteosarcoma
LDH: Lactate dehydrogenase
MR: Magnetic resonance
STS: Soft tissue sarcoma
TLS: Tumor lysis syndrome
US: Ultrasound
